# Silibinin Triggers the Mitochondrial Pathway of Apoptosis in Human Oral Squamous Carcinoma Cells

**DOI:** 10.31557/APJCP.2020.21.7.1877

**Published:** 2020-07

**Authors:** Radhika Murali Iyengar, Ezhilarasan Devaraj

**Affiliations:** *Department of Pharmacology, Biomedical Research Unit and Laboratory Animal Research Centre, Saveetha Dental College, Saveetha Institute of Medical and Technical Sciences, Saveetha University, Chennai, Tamil Nadu, India. *

**Keywords:** Silybum marianum, Cytochrome c, Caspases, Cytotoxicity, Apoptosis

## Abstract

**Background:**

Silibinin, a natural polyphenolic flavonoid present in seed extracts of milk thistle (Silybum marianum). It has been shown to interact with various cancer-related cell signalling pathways in preclinical models, demonstrating promising anticancer effects in vitro and in vivo.

**Materials and Methods:**

The cytotoxic effect of silibinin was evaluated in human oral squamous carcinoma (SCC-25) cells by MTT assay. The apoptosis-related morphological changes were investigated by AO/EB dual staining. The cytochrome c, caspases-3, and -9, B-cell lymphoma-2 (Bcl-2), and B-cell associated X protein (*Bax*) gene expressions were analysed by PCR.

**Results:**

We have shown that silibinin treatment for 24 h in SCC-25 cells induced cytotoxicity in a concentration-dependent manner. The cytotoxic potential was due to the induction of apoptosis via the release of mitochondrial cytochrome c into the cytosol and subsequent activation of caspases-3 and -9. Dual staining assay was further confirmed the induction of early apoptosis upon silibinin treatment.

**Conclusion:**

The results from this study show that silibinin can be considered as a promising drug candidate for the treatment of oral squamous cell carcinoma.

## Introduction

Oral cancer is the sixth most common cancer worldwide and a recent report pointed out that oral cancer is responsible for about 22% of cancer deaths worldwide. Oral squamous cell carcinoma (OSCC) accounts for 90% of all oral cancers (Attar et al., 2010). In India, oral cancer ranks in the top three of all cancers, which accounts for over 30% of all cancers reported in the country (Coelho, 2012). Tobacco use, including smokeless tobacco and excessive consumption of alcohol, is considered as the most important risk factors implicated in oral cancer-related death (Ezhilarasan et al., 2019). It has been reported that OSCC is a common malignant tumor of the head and neck and recurrence is an important prognostic factor in patients with OSCC (Wang et al., 2013). Among treatment modalities, surgery is the preferred treatment of OSCC. Despite great progress in chemo/radiotherapy, and targeted therapy, the prognosis of OSCC is poor due to aggressive local invasion and metastasis and recurrence. Thus, OSCC is still a challenging disease to treat (Li et al., 2015) and therefore, in view of the above scenario, there is an urgent need to develop a therapeutic drug candidate which target OSCC. 

Silymarin is a standardized hepatoprotective agent extracted and isolated from the medicinal plant silybum marianum (Milk thistle) (Ezhilarasan et al., 2014; Ezhilarasan, 2018). This extract contains well-defined ingredients including silibinin. Silibinin (SBN), a flavonolignane which represents approximately 60% of silymarin, has been identified as the major active agent (Šimánek et al., 2000; Ezhilarasan et al., 2016). In liver context, several studies have reported the anti-hepatoprotective effect of SBN against various drug and chemical-induced hepatotoxicity in experimental animals (Haddad et al., 2011; Ezhilarasan et al., 2012; Raghu et al., 2015; Ali et al., 2016; Ezhilarasan and Karthikeyan, 2016). However, ample evidence also suggests that SBN is not exclusively confined in the treatment of liver diseases, may function diversely and serve as a novel therapeutic drug candidate against liver, lung, prostate, colon, breast and, bladder cancers by regulating cancer cells growth, proliferation, apoptosis, angiogenesis and many other molecular mechanisms (Mateen et al., 2012; Gándara et al., 2014; Kil et al., 2014; Gu et al., 2015; Ezhilarasan et al., 2017). Silibinin has also been shown beneficial in clinical trials involving patients with prostate, breast and, liver cancers (Flaig et al., 2010; Bosch-Barrera et al., 2014). Recent studies have suggested that SBN is highly effective in combination with other drugs or compounds and can be used in the future treatment of cancer (Gu et al., 2015; Tsai et al., 2015). SBN has been demonstrated to induce apoptosis in various cancer cell types in vitro other than OSCC and in vivo by targeting multiple factors and influencing multiple signalling pathways. Hence, this study has been conducted to explore the anticancer efficacy of SBN against oral squamous cell carcinoma cell line (SCC-25) in vitro.

## Materials and Methods

*Chemicals*

Silibinin, 3-(4,5-dimethylthiazol-2-yl)-2,5-diphenyltetrazolium Bromide (MTT), dimethyl sulfoxide (DMSO) was purchased from Sigma Chemical Co. India. The other chemicals used in this study were purchased locally and were of analar grade.

*Cell culture*

The SCC-25 human oral squamous carcinoma cell line was procured from ATCC. Cells were maintained in Dulbecco’s Minimum Essential Media and Ham’s F-12 (1:1 ratio) supplemented with 10% fetal bovine serum (FBS), penicillin (100 units/ml) and streptomycin (100 μg/ml). Cells were cultured in a humidified atmosphere with 5% CO_2_ at 37 °C. Cells were grown in 75 cm^2 ^culture flasks and after a few passages, the cells were seeded for experiments. The experiments were done at 70 to 80% confluence. Upon reaching confluence, cells were detached using 0.05% Trypsin-EDTA solution. 

*Cell treatment*

Silibinin was dissolved in 0.1% DMSO (v/v). SCC-25 cells were plated at 10,000 cells/cm^2^. After 24 h, cells were fed with fresh expansion culture medium supplemented with different final concentrations of SBN (50 and 100 µM) or the corresponding volumes of the vehicle. The maximum concentration range employed in this study was fixed at 100 µM based on previously published literatures (Jiang et al., 2015; Tsai et al., 2015). At the end of the treatment period, cells were collected by trypsin application. The total cell number was determined by counting each sample in triplicate under the inverted microscope. 

*MTT assay*

Cytotoxic effect of SBN on SCC-25 cells was assessed by MTT assay (Safadi et al., 2003). Cells were seeded in 96-well plate at a concentration of 5 × 10^4^ cells/well. After 24 h, cells were fed with fresh expansion culture medium supplemented with different final concentrations of SBN (50 and 100 µM) and incubated for 24 h. Untreated cells served as control and vehicle control received only 0.1% DMSO. At the end of the treatment period, media from control, SBN treated cells were discarded and 50 μl of MTT (5 mg/ml of phosphate-buffer saline (PBS)) was added to each well. Cells were then incubated for 4 h at 37°C in CO_2_ incubator. MTT was then discarded and the coloured crystals of produced formazan were dissolved in 150 μl of DMSO and mixed effectively. The purple-blue formazan dye formed was measured using an ELISA reader (BIORAD) at 570 nm. 

*Acridine orange/ethidium bromide (AO/EB) Dual staining*

Acridine orange/ethidium bromide orange staining was carried out by a standard method (Gohel et al., 1999). SCC-25 cells were plated at a density of 1×10^4^ in 48-well plates. They were allowed to grow until they are 70–80% confluent. After 24 h, the cells were treated with different concentrations of SBN. The culture medium was aspirated from each well and cells were gently rinsed twice with PBS at room temperature. Then equal volumes of cells from control and SBN treated were mixed with 100 μl of dye mixture (1:1) of ethidium bromide and acridine orange and viewed immediately under Nikon inverted fluorescence microscope (Ti series, Tokyo, Japan) at 10x magnification. A minimum of 300 cells was counted in each sample in two different fields. The percentage of apoptotic cells was determined by [% of apoptotic cells = (total number of apoptotic cells/total number of cells counted) ×100].

*Gene expression analysis*

Total RNA was extracted by trizol reagent according to the standard protocol. The concentration of the extracted RNA was determined and the integrity of RNA was visualized on a 1% agarose gel using a gel documentation system (BioRad, Hercules, CA). The first strand of cDNA was synthesized from 1 μg of total RNA by reverse transcriptase using M-MLV (Promega, Madison, WI) and oligo (dT) primers (Promega) according to the manufacturer’s protocol. Then, 2 μl of template cDNA was added to the final volume of 20 μl of the reaction mixture. RT-PCR cycle parameters included 10 min at 95° C followed by 40 cycles involving denaturation at 95° C for 15 sec, annealing at 60° C for 20 sec, and elongation at 72°C for 20 sec. The sequences of the specific sets of primer for Bax, Bcl-2, cytochrome c, caspase-3, caspase-9 and GAPDH used in this study were taken from literatures. Expressions of selected genes were normalized to the *GAPDH* gene, which was used as an internal housekeeping control. All the RT-PCR experiments were performed in triplicate.

*Statistical analysis*

Data were expressed as mean ± S.E.M and analysed by Tukey’s test to determine the significance of differences between groups. A P value 0.01 or/and 0.001 was considered to be significant.

## Results

*Antiproliferative effect of SBN against human SCC-25 oral carcinoma cells*

**Figure 1:**
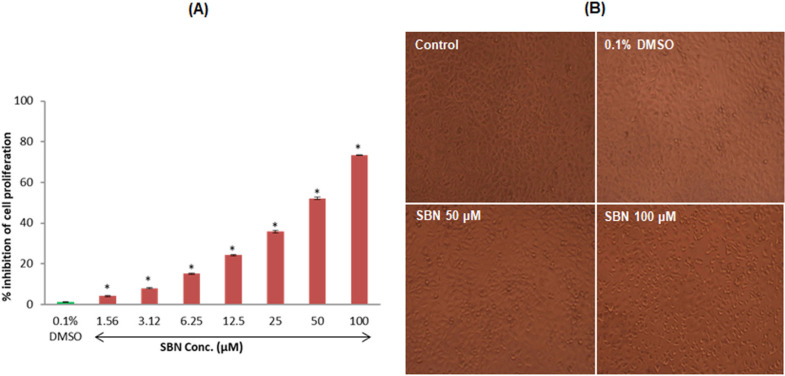
(A), Cytotoxic effect of SBN was analyzed by MTT assay. SCC-25 cells were treated with different concentrations of SBN for 24 h. Values are expressed as mean ± S.E.M. (n=3) *P < 0.001 vs control; (B), Morphology of control and SBN treated SCC-25 cells

We assessed the effect of SBN on the viability of SCC-25 cells. The cells were exposed to various concentrations of SBN for 24 h, and the cell viability was measured by MTT assay. Initially, we evaluated the cytotoxicity of SBN in logarithmic concentrations (1.56, 3.12, 6.25, 12.5, 25, 50 and 100 µM/ml) on SCC-25 cells. In this study, SBN caused a marked increase in cell death in a concentration-dependent manner. At the end of 24 h, maximum inhibition (73.42%) of cell growth was found at a maximum concentration (100 µM/ml) used in this study when compared to control. Further experiments were carried out with 50 and 100 µM/ml (Figure 1A). The DMSO treated cells did not produce any significant change in the viability of SCC-25 cells. The morphology of cells treated with SBN is presented in Figure 1B.

*AO/EB double-staining by fluorescent microscopy*

**Figure 2:**
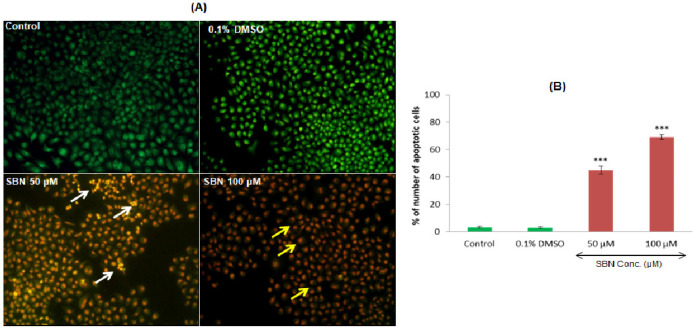
Apoptosis Related Morphological Changes (A) Acridine Orange/Ethidium Bromide Staining. White and yellow arrows indicating early and late apoptotic cells respectively (B) Quantification of apoptotic cell percentage. Values are expressed as mean ± S.E.M. (n=3). *** p < 0.001 vs control

Dual AO/EB fluorescent staining can detect basic morphological changes in apoptotic cells. In addition, it allows for the distinction between normal cells, early and late apoptotic cells, and necrotic cells. In this study, the negative control group (normal cells) and DMSO treated vehicle control group cells exhibit with the circular nucleus uniformly distributed in the center of the cell. While in the experimental groups, 50 µM/ml of SBN treatment caused early apoptosis and the cells were visualized as yellowish-green by AO nuclear staining. In higher concentration i.e., 100 µM/ml, SBN induced late apoptotic or cell death as evidenced by EB positive red-colored cells (Figure 2A). The percentage of apoptotic nuclei were increased (p<0.001) dose-dependently after SBN treatments in SCC-25 cells as compared to control (Figure 2B).

*Apoptotic marker gene expressions*

**Figure 3:**
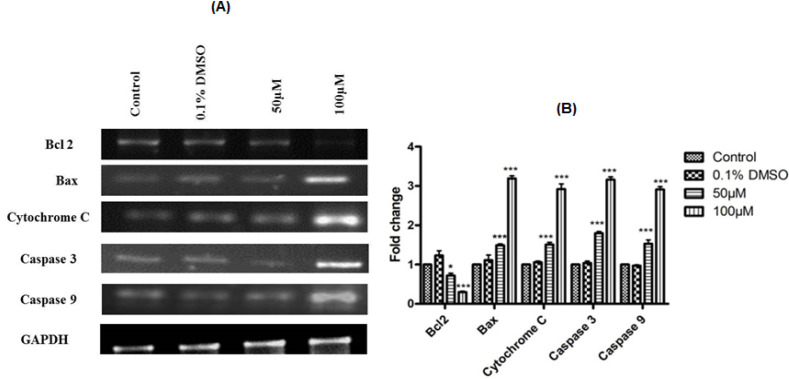
(A), Silibinin effect on the apoptotic marker genes expression; (B), Quantification of gene expression. Values are expressed as mean ± S.E.M (n=3). Comparisons were done by Bonferroni’s post-hoc comparison test. GAPDH used as an internal control for optimization. * p < 0.05; *** p < 0.001 vs control

To further confirm the apoptosis-inducing potentials of SBN at molecular level, we evaluated the gene expression analysis of pro and anti-apoptotic genes (*bax* and* bcl-2*), *cytochrome c*, *caspase 3 *and *9*. SBN treatments induced *caspases 3* and *9*, *bax* and *cytochrome c* gene expressions in dose-dependent manner as compared to control and DMSO treated cells. Further, SBN treatments also caused a significant downregulation of bcl-2 (an inhibitor of apoptosis) expression. All the observed effects were more prominent with high concentration of SBN used in this study i.e., 100 µM/ml (Figure 3A, B). In all cases, GAPDH used as an internal control for normalization.

**Figure 4:**
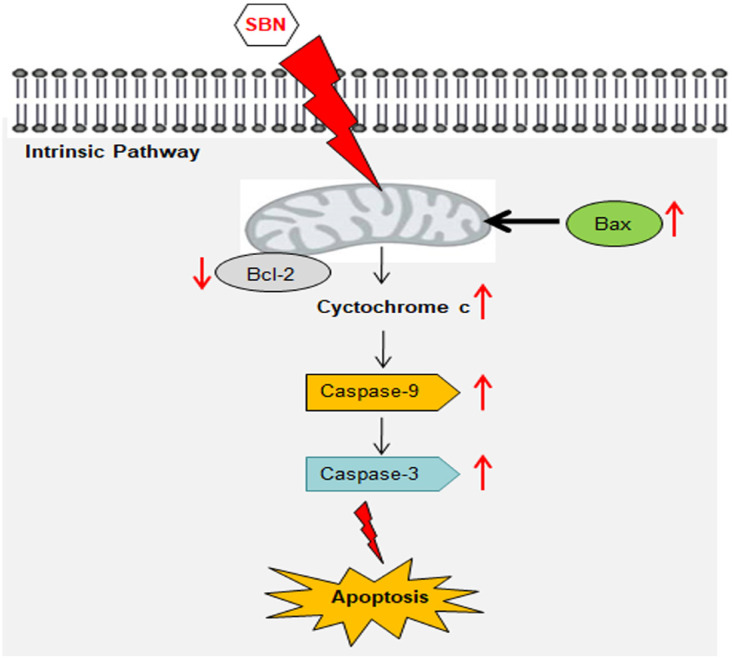
Possible Mechanism of Silibinin-Induced Apoptosis in Human Oral Squamous Cell Carcinoma SCC-25 Cell Lines

## Discussion

Silibinin has been widely used for the treatment of various hepatic ailments over 30 years (Feng et al., 2016; Ezhilarasan, 2018). In the liver context, SBN showed promising effect against hepatocellular carcinoma (Zhang et al., 2013; Li and Wang, 2016). SBN has prominent anticancer effects against lung, prostatic, colon, breast, bladder and liver cancer cell lines (Ezhilarasan, 2018; Mateen et al., 2012; Gándara et al., 2014; Kil et al., 2014; Gu et al., 2015). Although these studies imply that SBN has inhibitory effects on various cell lines and its influence on human oral squamous carcinoma cells (SCC-25) remains unclear. In the present study, SBN treatment caused cytotoxicity (73.42%) with maximum concentration used in this study (100 µM). In previous studies, SBN has been demonstrated for its antiproliferative effect against various cancer cell lines (Hagelgans et al., 2014; Bosch-Barrera and Menendez, 2015; Shukla et al., 2015; Jahanafrooz et al., 2016). In light of the above studies, the present result suggests that SBN can inhibit proliferation by inducing cytotoxicity regardless of cancer cell line and our current results are consistent with previous results. To further delineate the exact reason for the cytotoxic effect, we investigated the pro-apoptotic potentials of SBN in SCC-25 cells. 

Dual AO/EB fluorescent staining, visualized under a fluorescent microscope, can be used to identify apoptosis-associated changes of cell membranes during the process of apoptosis (Gherghi et al., 2003). This method can also accurately distinguish cells in different stages of apoptosis and hence commonly employed to detect apoptosis (Baskić et al., 2006; Liu et al., 2015). In this study, yellowish-green stained cells found upon SBN treatment indicate that SBN at low concentration can induce early apoptosis in SCC-25 cells. However, at higher concentration, SBN caused late apoptosis because EB staining only enters the cell with damaged membranes, such as late apoptotic and dead cells, emitting orange-red fluorescence when bound to concentrated DNA fragments or apoptotic bodies (Ribble et al., 2005). To further confirm mechanistically we evaluated the molecular parameters responsible for apoptosis induction.

Apoptosis is an orchestrated molecular event and plays a key role in the pathogenesis of various cancer. Hence, the induction of apoptosis is one of the underlying principles of most current cancer therapies (Shebi et al., 2019; Westhoff et al., 2016). The tumor cells may undergo several molecular mechanisms to suppress apoptosis and acquire resistance to apoptotic agents by the expression of an anti-apoptotic gene such as *bcl-2* or by the downregulation of pro-apoptotic gene such as *bax* (Hassan et al., 2014). The bcl-2 is compartmentalized in an outer membrane of mitochondria, where it plays an important role in promoting cell survival and inhibiting the actions of pro-apoptotic proteins. These pro-apoptotic proteins (bax) are in turn inhibited by the function of bcl-2 (Hardwick and Soane, 2013). In view of the above reports, it is clear that induction of pro-apoptotic (bax) and inhibition anti-apoptotic (*bcl-2*) gene expression can promote apoptosis in cancer cells and hence it is suggested as one of the therapeutic strategies in cancer treatment. In this study, SBN treatment caused a significant up regulation of pro-apoptotic *bax* gene expression and was well correlated with the downregulation of anti-apoptotic *bcl-2* expression and our current results are in agreement with the above reports.

Mitochondria is one of the key regulators of apoptosis, have shown to be involved in integrating different pro-apoptotic pathways via the release of cytochrome c into the cytosol (Ott et al., 2002). The pro-apoptotic gene in the *bcl-2* family is bax and it normally acts on the mitochondrial membrane to promote permeabilization and release of cytochrome c that is one of the important signals in the apoptosis cascade (Hardwick and Soane, 2013). The released cytochrome c is complexed with Apaf-1 and pro-caspase 9 in the cytosol forms apoptosome the release of activated caspases 9 and 3 further initiates the activation of caspase cascade leading to biochemical and morphological changes associated with apoptosis (Gheena and Ezhilarasan, 2019). These caspases are activated in response to a variety of cell death stimuli (Parrish et al., 2013; McIlwain et al., 2015). The present study shows that significant increase in* cytochrome c* gene expression upon SBN treatment in SCC-25 cells and this in turn responsible for the up regulation of caspases 9 and 3 which are downstream target of cytochrome c, therefore, release of cytochrome c from mitochondria is considered a key initial step in the apoptotic process (Figure 4). In light of these reports, it is suggested that the SBN treatment to SCC-25 cells could have caused the cytochrome c release in the cytoplasm due to the activation of pro-apoptotic gene *bax* that results in apoptosis via activation of caspase 9 and 3 cascade. 

In conclusion, the present study demonstrates the cytotoxic effect of SBN on SCC-25 cells and it was mainly due to the induction of apoptosis by triggering the mitochondrial pathway (activation of cytochrome c, caspases 9 and 3). Our molecular studies are well corroborated with dual staining. Hence, SBN may be considered as a potential drug candidate for treating oral squamous carcinoma.
